# PARP6 is a Regulator of Hippocampal Dendritic Morphogenesis

**DOI:** 10.1038/srep18512

**Published:** 2016-01-04

**Authors:** Jeffrey Y. Huang, Kang Wang, Anke Vermehren-Schmaedick, John P. Adelman, Michael S. Cohen

**Affiliations:** 1 Department of Physiology and Pharmacology, Oregon Health & Science University, Portland, Oregon 97210, United States; 2 Vollum Institute, Oregon Health & Science University, Portland, Oregon 97210, United States

## Abstract

Mono-ADP-ribosylation (MARylation) of mammalian proteins was first described as a post-translational modification catalyzed by bacterial toxins. It is now known that endogenous MARylation occurs in mammalian cells and is catalyzed by 11 members of the poly-ADP-ribose polymerase (PARP) family of proteins (17 in humans). The physiological roles of these PARPs remain largely unknown. Here we demonstrate that PARP6, a neuronally enriched PARP that catalyzes MARylation, regulates hippocampal dendrite morphogenesis, a process that is critical for proper neural circuit formation during development. Knockdown of PARP6 significantly decreased dendritic complexity in embryonic rat hippocampal neurons in culture and *in vivo*. Expression of wild-type PARP6 increased dendritic complexity; conversely, expression of a catalytically inactive PARP6 mutant, or a cysteine-rich domain deletion mutant that has significantly reduced catalytic activity, decreased dendritic complexity. The identification of PARP6 as a regulator of dendrite morphogenesis supports a role for MARylation in neurons during development.

The transfer of ADP-ribose from nicotinamide adenine dinucleotide (NAD^+^) to amino acids on proteins, a process known as mono-ADP-ribosylation (MARylation), is an ancient, reversible post-translational modification (PTM), discovered initially as the pathogenic mechanism of action of various bacterial toxins, such as diphtheria and cholera toxin^1^. Soon after these initial findings several studies provided evidence for the existence of bacterial toxin-independent endogenous MARylation in mammalian tissues, particularly the brain^2^,^3^,^4^,^5^,^6^. For example, Nestler and co-workers demonstrated that several proteins are MARylated in rat brain homogenates using ^32^P-labeled NAD^+4^. In another study, Tsuyama and co-workers purified four ~66 kilodalton (kDa) proteins from rat brain that catalyzed MARylation of actin^7^. While these studies demonstrate the existence of MARylation in the mammalian brain, neither the identity nor function of these putative endogenous MARylating enzymes was described.

A family of 17 proteins in humans known as poly-ADP-ribose polymerases (PARPs) share a conserved, diphtheria-toxin like catalytic domain^1^. This family is named after its founding member, PARP1, which catalyzes the formation of polymers of ADP-ribose, a PTM known as poly-ADP-ribosylation (PARylation). However, a recent study by Chang and co-workers revealed that most PARPs (i.e. PARP3, 4, 6-8, 10-12, 14-16) catalyze MARylation while only four PARPs (i.e. PARP1, 2, 5a, 5b) catalyze PARylation^8^. While much is known about the cellular roles of PARPs that catalyze PARylation, especially the role of PARP1 in the DNA damage response^9^,^10^, much less is known about the physiological roles of PARPs that catalyze MARylation (referred to here as mono-PARPs). Nevertheless, recent studies suggest that MARylation is more widespread in cell biology than previously appreciated^11^,^12^. Initially thought to be restricted to the nucleus, mono-PARPs are found throughout the cell and play roles in regulating cell viability, cell division, membrane structure, and the actin cytoskeleton^13^. However, the physiological functions of most mono-PARPs remain unknown.

Given the existence of endogenous MARylation in the mammalian brain, we sought to determine if any of the mono-PARPs play a physiological role in neurons. We found that PARP6 is one of the most abundantly expressed mono-PARPs in the developing mouse brain, and its expression peaked during a critical period of dendritic growth and branching in primary rat hippocampal neurons. We found that knockdown of PARP6 significantly reduced dendritic complexity in primary rat hippocampal neurons in culture and *in vivo*. Overexpression of wild type (WT) PARP6 significantly increased dendritic complexity whereas overexpression of catalytically inactive PARP6 or a cysteine-rich domain (CRD) deletion mutant significantly reduced dendritic complexity. Taken together, these results demonstrate that PARP6 has an essential role in neurodevelopment by regulating dendrite morphogenesis.

## Results

### PARP6 is highly expressed in the brain during development

**Figure 1:**
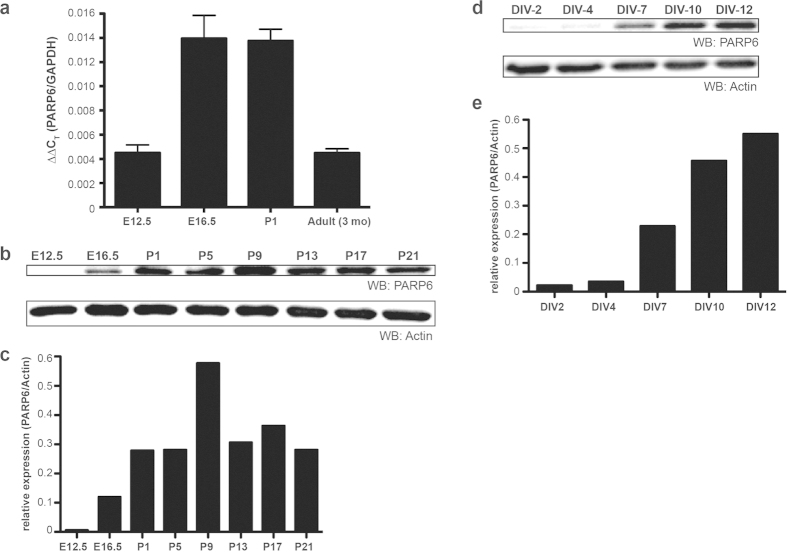
PARP6 is highly expressed in the developing rodent brain and primary hippocampal neurons. (**a**) PARP6 mRNA is present in the developing mouse brain. Quantitative real-time PCR of total mRNA from embryonic day E12.5 and E16.5, postnatal day P1, and adult (3 month old) mouse neocortex probed against PARP6-specific primers. Specific primers against GAPDH are used as an internal reference control for quantitative analysis by comparative C_T_ method. (n = 5, 6, 4, 5 for E12.5, E16.5, P1, and adult, respectively). Error bars represent standard error of the mean (SEM). (**b**) PARP6 protein is present from mid-embryonic to early postnatal stages of development in the mouse brain. Whole cell lysates were prepared from mouse neocortices harvested on E12.5, E16.5, P1, P5, P9, P13, P17, and P21. Proteins were resolved by SDS/PAGE and detected by Western blot with anti-PARP6 and anti-actin antibodies. (**c**) Quantification of PARP6 protein levels relative to actin in (**b**). (**d**) Endogenous PARP6 protein is detected in E18 primary rat hippocampal neurons grown in culture. Whole cell lysates were prepared from neurons harvested at different days *in vitro* (DIV). Proteins were resolved by SDS/PAGE and detected by Western blot with anti-PARP6 and anti-actin antibodies. (**e**) Quantification of PARP6 protein levels relative to actin in (**d**).

Previous studies support the existence of endogenous MARylation in the rodent brain, but the identity of the mono-PARPs that mediate this PTM in the brain are unknown. We first searched the Allen Brain Atlas^14^ and the EMAGE database^15^ to identify mono-PARP mRNAs that are expressed in the mouse nervous system. The mRNA for PARP6 is present at high levels in the adult mouse brain, particularly in the hippocampus, and is enriched in the nervous system in the embryonic day 14.5 (E14.5) mouse. Consistent with these data, we found that PARP6 mRNA was one of the most highly abundant mono-PARP mRNAs in E18 primary rat hippocampal neurons (Supplementary Fig. 1). We found that PARP6 mRNA levels in the mouse neocortex were highest from late embryonic (E16.5) to early postnatal (P1) stages (Fig. 1a). Next, we examined the levels of PARP6 protein expression in the mouse neocortex using an antibody against PARP6. The specificity of the antibody was established using two different methods: (1) lentiviral-mediated shRNA knockdown of PARP6 in neurons and (2) overexpression of HA-tagged PARP6 (HA-PARP6) in human embryonic kidney (HEK) 293T cells (Supplementary Fig. 2a,b). PARP6 protein levels were first detectable at E16.5 and peaked at P9 (Fig. 1b,c). In primary rat hippocampal neurons, PARP6 protein was first detectable at day *in vitro* 7(DIV7), and its levels progressively increased over time (Fig. 1d,e). Together, these demonstrate that PARP6 is an abundantly expressed, neuron-enriched mono-PARP during neurodevelopment.

### PARP6 is required for dendritic growth and maintenance in hippocampal neurons *in vitro*

**Figure 2:**
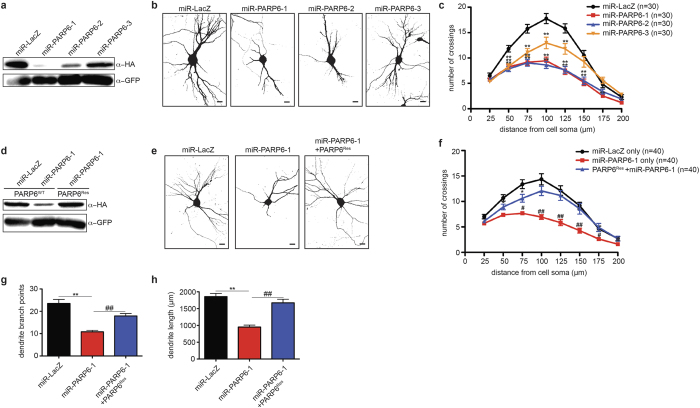
PARP6 regulates dendritic complexity in primary rat hippocampal neurons. (**a**) miRNA-based plasmids effectively knockdown PARP6 protein levels. HEK 293T cells were co-transfected with HA-PARP6 and GFP-expressing miRNA-based knockdown plasmids targeting rat PARP6 (miR-PARP6-1, 2, or 3) or LacZ (miR-LacZ). Proteins were resolved by SDS/PAGE and detected by Western blot with anti-HA and anti-GFP antibodies. (**b**) Knockdown of PARP6 using miRNA-based plasmids decreased dendritic complexity. E18 primary rat hippocampal neurons were transfected with miRNA-based knockdown plasmids on DIV7 and fixed on DIV12. Shown are representative binary images generated from fluorescent images. Scale bar, 20 μm. (**c**) Quantification of results in (**b)** using Sholl analysis. Error bars represent SEM. *p < 0.05, **p < 0.001 (one-way ANOVA followed by Tukey’s HSD test) compared to miR-LacZ. (**d**) PARP6^Res^ is resistant to knockdown by miR-PARP6-1. HEK 293T cells were co-transfected with HA-PARP6^WT^ or HA-PARP6^Res^. Proteins were resolved by SDS/PAGE and detected by Western blot with anti-HA and anti-GFP antibodies. (**e**) PARP6^Res^ rescues miR-PARP6-1-mediated decrease in dendritic complexity. E18 primary rat hippocampal neurons were transfected either with miR-LacZ, miR-PARP6-1, or miR-PARP6-1 and HA-PARP6^Res^ on DIV7 and fixed on DIV12. Shown are representative binary images generated from fluorescent images. Scale bar, 20 μm. (**f**) Quantification of results in **e** using Sholl analysis. Error bars represent SEM. *p < 0.05, **p < 0.001 (one-way ANOVA followed by Tukey’s HSD test) compared to miR-LacZ only. ^#^p < 0.05, ^##^p < 0.001 (one-way ANOVA followed by Tukey’s HSD test) compared to PARP6^Res^ + miR-PARP6-1. (**g**) PARP6^Res^ restores miR-PARP6-1-mediated decrease in dendritic branching. Quantification of total number of dendrite branch points from neurons described in error bars represent SEM. **p < 0.001 (one-way ANOVA followed by Tukey’s HSD test) compared to miR-LacZ; ^##^p < 0.001 (one-way ANOVA followed by Tukey’s HSD test) compared to miR-PARP6-1. (**h**) PARP6^Res^ restores miR-PARP6-1-mediated decrease in dendritic length. Quantification of total dendrite length from neurons described in (**e)**. Error bars represent SEM. **p < 0.001 (one-way ANOVA followed by Tukey’s HSD test) compared to miR-LacZ; ^##^p < 0.001 (one-way ANOVA followed by Tukey’s HSD test) compared to miR-PARP6-1.

PARP6 expression in primary rat hippocampal neurons occurs during a critical period of dendrite morphogenesis where there is an extensive amount of dendritic growth and branching^16^. To determine if PARP6 plays a role in dendrite morphogenesis in rat hippocampal neurons, we used Pol II-driven miRNA^17^ plasmids to knockdown endogenous rat PARP6. These constructs also express GFP, which is essential for identifying transfected cells. We first determined the efficiency of three miRNA-based knockdown plasmids targeting rat PARP6 (miR-PARP6-1, 2, and 3) in HEK 293T cells expressing rat HA-PARP6. We found that all three miR-PARP6 constructs effectively knocked down HA-PARP6 protein levels compared to a control miRNA-based knockdown construct (miR-LacZ), with miR-PARP6-1 being the most potent (Fig. 2a).

To determine if knockdown of PARP6 affects dendritic complexity, we transfected rat hippocampal neurons with miR-PARP6-1, 2, 3, or miR-LacZ on DIV7 and fixed neurons on DIV12. We found that transfection with miR-PARP6-1, 2, and 3 resulted in a significant reduction in dendritic complexity as determined by Sholl analysis (Fig. 2b,c). The degree of the decrease in dendritic complexity directly correlated with potency of the miR-PARP6 constructs, supporting the notion that these effects are due to PARP6 knockdown (Fig. 2a–c). To further confirm the specificity of PARP6 knockdown, we designed a PARP6 expression construct that is resistant to the miR-PARP6-1 target sequence (PARP6^Res^) (Fig. 2d). We found that PARP6^Res^ was able to restore the miR-PARP6-1-mediated reduction in dendritic complexity (Fig. 2e,f) Further quantification demonstrated that PARP6 knockdown decreased both dendritic branching and length, which were restored by PARP6^Res^ (Fig. 2g,h).

The reduction in dendritic complexity in PARP6 knockdown neurons could be due to a decrease in dendritic growth and/or maintenance. To address this issue, we examined the effects of PARP6 knockdown on dendritic complexity in hippocampal neurons at two different time points in culture. Hippocampal neurons were transfected with miR-LacZ or miR-PARP6-1 on DIV6 and subsequently fixed on DIV7 or DIV12. In neurons transfected with miR-LacZ, dendritic complexity increased from DIV7 to DIV12; by contrast, dendritic complexity decreased from DIV7 to DIV12 in miR-PARP6-1 transfected neurons (Supplementary Fig. 3a,b). Taken together, these results demonstrate that PARP6 regulates both dendritic growth and maintenance in hippocampal neurons.

### PARP6 regulates hippocampal dendritic complexity *in vivo*

We next sought to examine the effects of PARP6 knockdown on dendritic complexity in rat hippocampal neurons in an *in vivo* context, where neurons are subject to extrinsic factors that regulate dendrite morphogenesis that are not present in cultured neurons^18^. To knockdown PARP6 in hippocampal neurons *in vivo*, we used *in utero* electroporation to introduce GFP-expressing shRNA plasmids that target PARP6 (shPARP6-1,2). Both shRNA plasmids knockdown HA-PARP6 protein levels in HEK293T cells; shPARP6-1 was more potent than shPARP6-2 (Supplementary Fig. 4a). Consistent with our results using a miRNA-based knockdown strategy, knockdown of PARP6 with either shPARP6-1 or shPARP-2 significantly decreased dendritic complexity compared to an shRNA control (shControl) (Supplementary Fig. 4b,c). As expected based on their potencies, shPARP6-1 demonstrated a more pronounced reduction in dendrite complexity, while the less potent shPARP6-2 showed an intermediate reduction in dendrite complexity (Supplementary Fig. 4).

**Figure 3:**
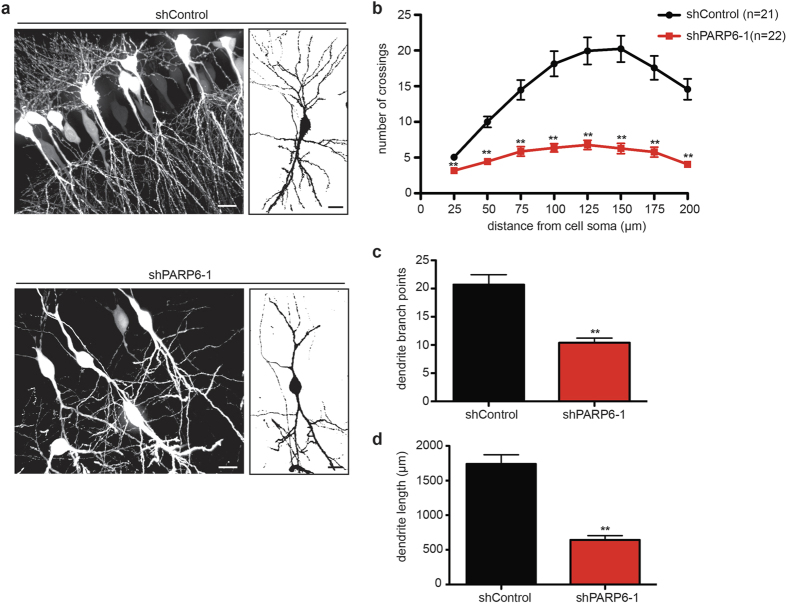
PARP6 regulates dendritic complexity in hippocampal neurons *in vivo.* (**a**) Knockdown of PARP6 *in vivo* with a GFP-expressing shRNA-based plasmid targeting PARP6 (shPARP6-1) decreased dendritic complexity in CA1 hippocampal neurons. *In utero* electroporation using either shPARP6-1 or shControl was performed on E19 timed-pregnant rats. Brains were fixed on P14 and sections cut into 100 μm-thick coronal slices by vibratome sectioning. Shown are representative fluorescent (left) and binary (right) images of rat CA1 hippocampal neurons. Scale bar, 20 μm. (**b**) Quantification of results in a using Sholl analysis. Error bars represent SEM. **p < 0.001 (two-tailed paired t-test). (**c**) Knockdown of PARP6 with shPARP6-1 decreased dendritic branching in hippocampal neurons *in vivo*. Quantification of total number of dendrite branch points from neurons described in (**a**). Error bars represent SEM. **p < 0.001 (two-tailed paired t-test). (**d**) Knockdown of PARP6 with shPARP6-1 decreased dendritic length in hippocampal neurons *in vivo*. Quantification of total dendrite length from neurons described in (**a**). Error bars represent SEM. **p < 0.001 (two-tailed paired t-test).

To test the *in vivo* effects of PARP6 knockdown in hippocampal neurons, we delivered either shPARP6-1 or shControl into E19 rat hippocampi using *in utero* electroporation^19^. *In utero* electroporated rat brain tissue was collected on postnatal day 14 (P14) and shRNA-expressing CA1 hippocampal neurons were identified by GFP expression. Knockdown of PARP6 *in utero* significantly reduced dendrite complexity (Fig. 3a,b) and total dendritic branch points and length (Fig. 3c,d) in P14 rat CA1 hippocampal neurons. Taken together, these results demonstrate that PARP6 regulates dendritic growth and branching in CA1 hippocampal neurons *in vivo*.

### The MARylation activity of PARP6 is required to promote dendrite complexity in hippocampal neurons *in vitro*

**Figure 4:**
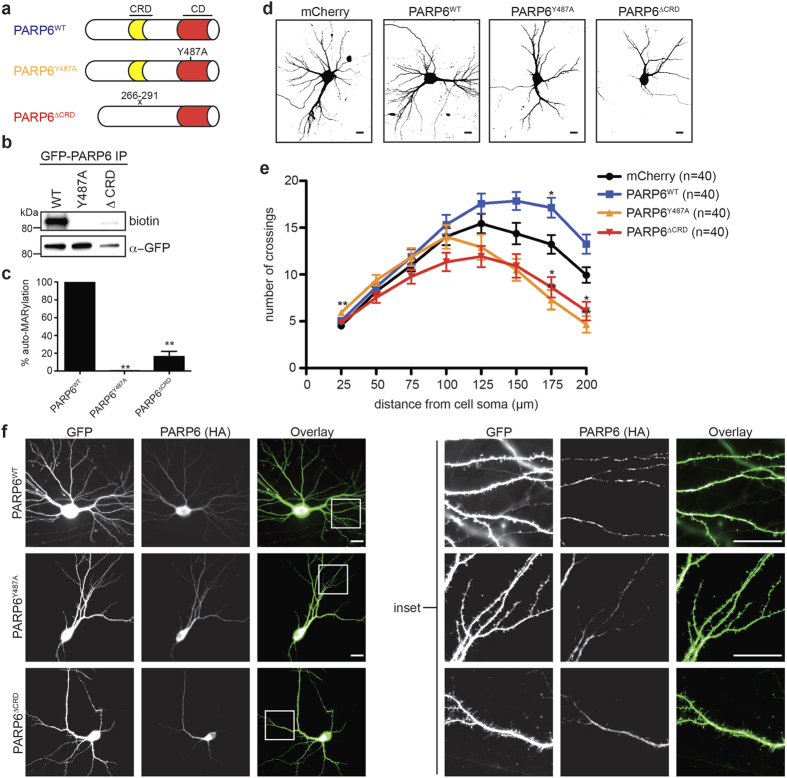
Ablation of PARP6 catalytic activity or deletion of a conserved cysteine-rich domain decreases dendritic complexity in primary hippocampal neurons. (**a**) Schematic architecture of PARP6^WT^, PARP6^Y487A^, and PARP6^ΔCRD^. CRD = cysteine-rich domain, which is conserved in all PARP6 orthologues. (**b**) PARP6^Y487A^ is inactive and PARP6^ΔCRD^ has significnatly reduced catalytic activity compared to PARP6^WT^. HEK 293T cells were transfected either with GFP-tagged PARP6^WT^, PARP6^Y487A^, or PARP6^ΔCRD^. Whole cell lysates were prepared and PARP6 proteins were immunoprecipitated using a GFP Nano-trap (Chromotek). Auto-ADP-ribosylation assays were performed on immunoprecipitated GFP-PARP6 variants using 6-alkyne modified NAD^+^(6-a-NAD^+^). Click chemistry was subsequently performed with biotin-azide and proteins were resolved by SDS/PAGE and detected by Western blot with anti-GFP or streptavidin-HRP. (**c**) Quantification of PARP6 auto-MARylation levels (biotin) in (**b**), normalized to GFP levels. Data presented as average of three independent experiments. Error bars represent SEM. **p < 0.001 (one-way ANOVA followed by Tukey’s HSD test) compared to GFP-PARP6^WT^. (**d**) PARP6^WT^ increased, whereas PARP6^Y487A^ and PARP6^ΔCRD^ decreased dendritic complexity in primary hippocampal neurons. E18 primary rat hippocampal neurons were co-transfected with GFP and either mCherry (control), HA-PARP6^WT^, HA-PARP6^Y487A^, or HA-PARP6^ΔCRD^ on DIV7 and fixed on DIV12. Shown are representative binary images generated from fluorescent images. Scale bar, 20 μm. (**e**) Quantification of results in (**d**) using Sholl analysis. Error bars represent SEM. *p < 0.05, **p < 0.001 (one-way ANOVA followed by Tukey’s HSD test) compared to mCherry. (**f**) HA-PARP6 exhibits a punctate distribution in dendrites of primary rat hippocampal neurons, which is unchanged by ablating catalytic activity or deleting the CRD. E18 primary rat hippocampal neurons were co-transfected with GFP and either HA-PARP6^WT^, PARP6^Y487A^, or PARP6^ΔCRD^ on DIV7 and fixed on DIV12. Neurons were immunolabeled with an anti-HA antibody. Scale bar, 20 μm.

A recent study showed that all mono-PARPs, including PARP6, have auto-MARylation activity^8^. We therefore sought to determine if the MARylation activity of PARP6 is necessary for promoting dendritic complexity in rat hippocampal neurons *in vitro*. We generated a GFP-PARP6 mutant, in which a conserved tyrosine (Tyr487, mouse numbering) was mutated to an alanine (GFP-PARP6^Y487A^), which is predicted to eliminate the MARylation activity of PARP6 (Fig. 4a)^1^. To examine the catalytic activity of PARP6, we developed a GFP-immunoprecipitation (IP)-auto-MARylation assay^8^ that uses 6-alkyne-NAD^+^(6-a-NAD^+^)^20^,^21^, a NAD^+^ analogue that can be coupled to biotin-azide via the copper(I) catalyzed [3 + 2] cycloaddition reaction (commonly referred to as the “click” reaction). In this assay GFP-PARP6 is immunoprecipitated using GFP Nano-Trap magnetic particles and auto-ADP-ribosylation is initiated by the addition of 6-a-NAD^+^. The magnetic particles are then subjected to click reaction conditions with biotin-azide, allowing detection of auto-MARylated PARP6. As expected, GFP-PARP6^WT^ exhibited robust auto-MARylation activity whereas GFP-PARP6^Y487A^ was completely inactive (Fig. 4b,c). These results confirm that PARP6 is an active mono-PARP and that Y487 is required for auto-MARylation activity.

Next, we examined the effects of overexpressing WT and catalytically inactive PARP6 on dendritic complexity in primary rat hippocampal neurons. Rat hippocampal neurons were transfected on DIV7 with either HA-PARP6^WT^ or HA-PARP6^Y487A^ together with GFP, which was used for identifying transfected neurons and analyzing dendritic complexity, and fixed on DIV12. We found that overexpression of HA-PARP6^WT^ significantly increased distal (175 μm from cell soma) dendritic complexity compared to a mCherry control (Fig. 4d,e). Conversely, overexpression of HA-PARP6^Y487A^ significantly decreased distal dendritic complexity (175 and 200 μm from cell soma) compared to a mCherry control, which suggests that catalytically inactive PARP6 mutant acts as a dominant negative (Fig. 4d,e). This dominant negative effect was not due to differences in localization between the WT and catalytically inactive PARP6: both HA-PARP6^WT^ and HA-PARP6^Y487A^ are found throughout the soma and distributed in a punctate pattern in dendrites in a similar manner (Fig. 4f). Taken together, these results demonstrate the necessity of PARP6 catalytic activity to promote dendritic complexity and further support the role of PARP6 as a regulator of dendrite morphogenesis.

### A PARP6 cysteine-rich domain (CRD) deletion mutant has significantly reduced auto-MARylation activity and acts as a dominant negative to decrease dendritic complexity

PARP6 contains a CRD (amino acids 266–291, mouse numbering) (Fig. 4a) that is just n-terminal to the catalytic domain^22^. The fact that this CRD is conserved in all PARP6 orthologues suggests that it is important for function (Supplementary Fig. 5). We therefore sought to determine if the CRD is necessary for PARP6 function in neurons. We first assessed the catalytic activity of a GFP-PARP6 deletion mutant lacking the CRD (GFP-PARP6^ΔCRD^). Using the GFP-IP-auto-MARylation assay, we found that GFP-PARP6^ΔCRD^ exhibited a ~6-fold decrease in MARylation activity compared to GFP-PARP^WT^ (Fig. 4b,c). Similar to catalytically inactive PARP6, overexpression of HA-PARP6^ΔCRD^ in rat hippocampal neurons significantly decreased distal dendritic complexity compared to a mCherry control (Fig. 4d,e). Deletion of the CRD did not affect PARP6 localization; there was no observable difference between the expression pattern of PARP6^WT^ and PARP6^ΔCRD^ in dendrites (Fig. 4f). Taken together, these results support a role for the CRD in regulating PARP6 function in neurons.

## Discussion

MARylation is emerging as an essential post-translational modification in cells; however, it is far less understood compared to other post-translational modifications, such as phosphorylation. The majority of PARP family members catalyze MARylation^8^, but we have limited knowledge of the physiological roles of these mono-PARPs. In this study we identified a role for the mono-PARP, PARP6, in neurodevelopment. We found that PARP6 is enriched in the nervous system during development and is the most abundantly expressed mono-PARP in E18 primary rat hippocampal neurons. Knockdown of PARP6 using two different strategies (miRNA- and shRNA-based) decreased dendritic growth and branching in primary rat hippocampal neurons *in vitro* and *in vivo*. Overexpression of catalytically inactive PARP6 or a cysteine-rich domain (CRD) deletion mutant decreased dendritic complexity. Taken together, these results demonstrate a role for PARP6 in regulating dendrite morphogenesis in rat hippocampal neurons during development.

We provide evidence that the MARylation activity of PARP6 is required for its role in regulating dendritic complexity in primary rat hippocampal neurons. This was demonstrated by the finding that overexpression of PARP6^WT^ increased dendritic complexity whereas overexpression of the catalytically inactive mutant PARP6^Y487A^ decreased dendritic complexity (Fig. 4d,e). While all mono-PARPs catalyze auto-MARylation^8^, they can also MARylate other proteins (in some cases, to a greater extent than auto-MARylation). Except in a few cases^11^,^23^, the protein substrates of individual mono-PARPs are unknown. This is due to the inability to identify direct protein substrates for individual PARPs in a cellular context. We have developed a strategy^20^ to overcome this limitation and are extending this strategy for identifying the direct substrates of PARP6 that will provide insight into the mechanism by which PARP6 regulates dendritic complexity.

The observation that overexpression of a CRD deletion mutant (PARP6^ΔCRD^) in hippocampal neurons acts as a dominant negative — decreasing dendritic complexity (Fig. 4d,e) — demonstrates the importance of the CRD for PARP6 function. Based on the distribution of cysteines in the CRD in PARP6 it was suggested that the CRD contains a putative zinc finger (ZnF) motif of the CCCH- or CCCC-type^22^. Interestingly, this ZnF motif is also found in the closely related paralogue PARP8^22^ (Supplementary Fig. 5). Originally identified as DNA binding motifs, ZnFs can also bind RNA, protein, and lipid substrates^24^.

The pronounced decrease in auto-MARylation activity of PARP6^ΔCRD^ (Fig. 4d,e) suggests that either the CRD is required for catalytic activity or the CRD itself contains site(s) of auto-MARylation. A previous study by Chang and co-workers identified sites of auto-MARylation in human PARP6 and PARP8^8^, but none of these were found in the CRD; however, this does not rule out that auto-MARylation does not occur within the CRD, especially given the challenges in identifying MARylation sites in proteins. To further dissect the role of the CRD in PARP6 function, it will be important to identify *bona fide* substrates of PARP6 in neurons.

Unlike the well-studied PTMs phosphorylation and ubiquitylation, the physiological functions of MARylation largely remain a mystery. Our study establishes the first potential function for MARylation in neurodevelopment: regulation of dendrite morphogenesis by the mono-PARP PARP6. A growing body of evidence suggests that defects in dendrite morphogenesis contribute to the pathogenesis of several neurodevelopmental disorders, such as autism and Rett’s syndrome. Elucidating the mechanism by which PARP6-mediated MARylation controls dendritic complexity will not only provide new insight into how dendrite morphogenesis is regulated during development, but could also lead to new therapeutic approaches for neurodevelopmental disorders.

## Materials and Methods

The use and care of animals used in this study follows the guidelines of the OHSU Institutional Animal Care and Use Committee. The preparation of rat hippocampal neurons and *in utero* electroporations in timed-pregnant rats were approved by the OHSU Institutional Animal Care and Use Committee.

### Preparation and culturing of primary hippocampal neurons

Hippocampi were dissected from the brains of E18 Sprague-Dawley rats (Charles River Laboratories), trypsinized, and dissociated with 0.25% trypsin (Gibco) and 1 mg ml^−1^ DNase (Roche). Primary neurons were plated at a density of 250,000 cells per 12-mm glass coverslip (Assistent) coated with 0.01% poly-D-lysine (Trevigen). Neurons were plated with plating media [NeuralQ^TM^ Basal medium (GlobalStem) supplemented with 10% fetal bovine serum (HyClone), 2 mM Glutamax (Gibco), 1 mM sodium pyruvate (Gibco), 100 IU penicillin and 100 μg mL^−1^ streptomycin (Gibco)] and grown in serum-free culture media [NeuralQ^TM^ Basal medium supplemented with GS21 (GlobalStem), 2 mM Glutamax, 100 IU penicillin and 100 μg mL^−1^ streptomycin]. Primary neurons were maintained at 37 °C in a humidified tissue-culture incubator containing 5% CO_2_. On day *in vitro* (DIV) 4, one-half of media was replaced with culture media containing 20 μM 5-fluorodeoxyuridine (FdU) (Acros Organics) to inhibit non-neuronal cell proliferation. Following FdU treatment, one-third of culture media was replaced with fresh culture media every 3 days.

### Primary neuron transfection

DIV6 or DIV7 hippocampal neurons were transfected with plasmid DNA using Lipofectamine 2000 (Invitrogen). For single plasmid transfections, 0.9 μg plasmid DNA was combined with 1.6 μL Lipofectamine 2000 in 100 μL total volume of NeuralQ^TM^ Basal. For two plasmid co-transfections, 1 μg total plasmid DNA was combined with 1.78μL Lipofectamine 2000 in 100 μL total volume NeuralQ^TM^ Basal. Lipofectamine-2000 reaction mixtures were incubated for 30 min at room temperature. One-half of culture media was removed and saved for later use as conditioned culture media. Reaction mixtures were added dropwise to cells and incubated at 37 °C/5%CO_2_ for 4 h. Transfection media was replaced with one-half conditioned culture media and one-half fresh culture media and cells were returned to the 37 °C/5% CO_2_ incubator until further processing.

### PARP family endpoint PCR profiling

Hippocampal tissue was dissected from E18 Sprague-Dawley rats and mRNA was isolated using TRIzol reagent (Invitrogen) and reverse-transcribed with SuperScript III reverse transcriptase (Invitrogen) to produce cDNA. Hippocampal cDNA was amplified using TAQ DNA polymerase with ThermoPol buffer (New England Biolabs) and rat PARP-specific primers (see Supplementary Table 1 for sequences) according to the following program: 95 °C – 30 sec; 35 cycles of 95 °C −30 sec, 63 °C −30 sec and 68 °C – 30 sec; 68 °C – 5 min. Hypoxanthine-guanine phosphoribosyltransferase (HPRT) was used as an internal reference gene. HPRT-specific primer sequences are as follows: 5′-GGTGAAAAGGACCTCTCGAAG-3′ (forward, bp 558–578) and 3′-GCTTTTCCACTTTCGCTGATG-5′ (reverse, bp 707–687). PCR products were run on a 2% agarose gel containing 0.2 μg mL^−1^ ethidium bromide. Relative levels of PARP gene expression were determined using densitometric quantitation of PARP expression normalized to HPRT internal reference gene expression. All graphs were generated using GraphPad Prism.

### Quantitative real-time PCR

Neocortices were dissected from the brains of E12.5, E16.5, P1, and 3 month-old (adult) C57BL/6 mice. mRNA was isolated from samples using TRIzol reagent and reverse-transcribed with SuperScript III reverse transcriptase to produce cDNA. Neocortical cDNA was amplified in duplicate by qRT-PCR using a SYBR Green detection reagent (Power SYBR Green, Applied Biosystems) and murine PARP6: 5′-CATTCTACTGCTCACCCCAAG-3′ (forward, bp 157-176) and 3′-ACCGTAGCCTCAACACAATAG-5′ (reverse, bp 298-278) or GAPDH: 5′-TGAACGGGAAGCTCACTGGCA-3′ (forward, bp 757-777) and 3′-TCAGATGCCTGCTTCACCACC-5′ (reverse, bp 886-866) specific primers according to the following program: 50 °C − 2 min; 95 °C − 10 min; 40 cycles of 95 °C − 15 sec then 60 °C − 1 min. GAPDH was used as an internal reference gene. Relative quantification of PARP6 gene expression was measured by the comparative ΔC_t_ method. All graphs were generated using GraphPad Prism.

### Immunoblot

Neocortices were dissected from the brains of E12.5, E16.5, P1, P5, P9, P13, P17, and P21 C57BL/6 mice, snap frozen in liquid nitrogen, and stored at −80 °C. Frozen neocortical tissue was lysed in tissue protein lysis buffer (50 mM Tris-HCl [pH 7.4], 150 mM NaCl, 1% Triton X-100) with cOmplete^TM^ protease inhibitors (Roche). Samples were homogenized using a BeadBug stainless steel bead homogenizer (Benchmark Scientific) at 4000× speed for 20 sec. Lysates were centrifuged at 10,000 × g for 5 min at 4 °C and supernatants were transferred to new sample tubes.

Primary hippocampal neurons were cultured on a PDL-coated 6-well plate (35 mm well^−1^, Greiner Bio-One) and harvested at DIV-2,4,7,10, and 12. Samples were collected by first washing cells in cold PBS, then lysing cells in total protein lysis buffer (50 mM Tris-HCl [pH 7.4], 150 mM NaCl, 1% Triton X-100) with cOmplete^TM^ protease inhibitors (Roche). Lysates were centrifuged at 3,000 × g for 5 min at 4 °C and supernatants were transferred to new sample tubes. Total protein concentration was determined by Bradford protein assay (Bio-Rad). 10 μg of total protein lysates in Laemmli sample buffer with 5% β-mercaptoethanol were heated at 95 °C for 5 min. Samples were resolved by SDS-PAGE and transferred onto 0.45 μm PVDF membranes (BioTrace™, Pall). Membrane blots were blocked with 5% milk-TBST for 1 h at room temperature. Blots were then probed with primary antibodies for 2 h at room temperature and HRP-conjugated secondary antibodies for 1 h at room temperature. ECL HRP substrate (SuperSignal^TM^ West Pico, ThermoFisher) was added to detect protein targets by chemiluminescence. Blots were imaged for chemiluminescent signal on a ChemiDoc MP system (Bio-Rad).

### Antibodies

The following primary antibodies were used for this study: anti-PARP6 (rabbit polyclonal, Sigma Prestige® HPA026991, 1:1000); anti-GAPDH (mouse monoclonal, Santa Cruz Biotechnology clone 6C5, 1:5000); anti-β-actin (mouse monoclonal, GenScript THE^TM^ clone 2D1D10, 1:1000); anti-HA (mouse monoclonal, Covance clone 16B12, 1:1000); anti-GFP (chicken polyclonal, Abcam ab13970, 1:5000); HRP-conjugated streptavidin (Jackson ImmunoResearch, 0.4 μg mL^−1^). All primary antibodies were diluted to working concentrations in 3% bovine serum albumin in TBST for immunoblot or 10% horse serum/2% bovine serum albumin in PBST (10% HS/2% BSA-PBST) for immunofluorescence.

The following secondary antibodies were used: HRP-conjugated goat anti-mouse IgG (Invitrogen, 1:5000), HRP-conjugated goat anti-rabbit IgG (Invitrogen, 1:5000), HRP-conjugated goat anti-chicken IgY (Santa Cruz Biotechnology, 1:5000), Alexa Fluor 488-conjugated donkey anti-mouse IgG (Molecular Probes, 1:1500), Cy3-conjugated donkey anti-mouse IgG (Jackson ImmunoResearch, 1:1000). All secondary antibodies were diluted to working concentrations in 5% milk-TBST for immunoblot or 10% HS/2% BSA-PBST for immunofluorescence.

### PARP6 RNA interference constructs

RNAi experiments were performed with either three SIBR vectors^17^ (miR-PARP6) – RNA polymerase II expression vectors expressing synthetic microRNAs – or two shRNA-expressing vectors (shPARP6) targeting the coding sequence of *Rattus norvegicus* PARP6 mRNA (GenBank accession no. NM_001106828) (see Supplementary Table 2 for RNAi sequences). RNAi constructs were designed using iRNAi version 2.1 software (Nucleobytes). Rat nucleotide BLAST database searches confirmed specificity of each target sequence. A nontargeting microRNA-expressing (miR-LacZ) or shRNA-expressing (shControl) vector was used as a negative control. All microRNA sequences were subcloned into the SIBR vector and all shRNA sequences were subcloned into a pUC vector containing a U6 promoter driving shRNA expression and a human EF1-alpha promoter driving GFP expression (gift from Anthony P. Barnes, OHSU). All sequences were verified by DNA sequencing.

All RNAi constructs were tested for PARP6 knockdown efficiency by co-transfecting HEK293T cells with RNAi-expressing plasmid and a PARP6 expression plasmid containing an N-terminus HA epitope sequence. HEK293T cells were transfected using CalPhos^TM^ mammalian transfection kit (Clontech). Cells were collected 48 h post-transfection and lysed in total protein lysis buffer (50 mM Tris-HCl [pH 7.4], 150 mM NaCl, 1% Triton X-100) with cOmplete^TM^ protease inhibitors. Lysates were centrifuged at 3,000 × g for 5 min at 4 °C and supernatants were transferred to new sample tubes. Total protein lysates were immunoblotted for PARP6 expression using a primary antibody against HA (Covance, HA.11 Clone 16B12) and for RNAi expression using a primary antibody against GFP (Abcam, ab13970). Relative PARP6 protein levels were determined using densitometric quantitation of PARP6 (HA) expression normalized to RNAi (GFP) expression. All graphs were generated using GraphPad Prism.

### PARP6 expression constructs

Wild-type PARP6 expression constructs were prepared by PCR-amplifying PARP6 from a mouse or rat whole brain cDNA library using PARP6-specific primers containing complementary restriction enzyme digest sequences for subcloning. PCR-amplified PARP6 was subcloned into a CAG vector containing either an N-terminus HA epitope sequence (HA-PARP6^WT^) or an N-terminus GFP sequence (GFP-PARP6^WT^) for immunofluorescence or immunoprecipitation.

A PARP6 rescue construct (PARP6^Res^) was designed using a GeneArt Strings (Life Technologies) gene fragment with three silent point mutations in the seed region (nucleotides 2–8) of the miR-PARP6-1 target sequence. The miR-PARP6-1-resistant gene fragment was PCR amplified with primers containing complementary restriction enzyme digest sequences for subcloning into the HA-PARP6^WT^ vector. Following PCR amplification, the non-resistant miR-PARP6-1 sequence of PARP6^WT^ was removed and replaced with the synthesized miR-PARP6-1-resistant gene fragment.

A PARP6 catalytically inactive mutant (PARP6^Y487A^) was constructed by site-directed mutagenesis of the HA-PARP6^WT^ or GFP-PARP6^WT^ vector using QuikChange II Site-Directed Mutagenesis Kit (Agilent Technologies) according to manufacturer’s protocol. PARP6^Y487A^ contains a single point-mutation at a key tyrosine residue (Y487A) in the PARP6 catalytic triad motif (H-Y-I).

A PARP6 cysteine-rich domain deletion mutant (PARP6^ΔCRD^) was designed using a GeneArt Strings (Life Technologies) gene fragment lacking a cysteine-rich domain spanning amino acids 266–291. The PARP6^ΔCRD^ gene fragment was PCR amplified with primers containing complementary restriction enzyme digest sequences for subcloning into the HA-PARP6^WT^ or GFP-PARP6^WT^ vector. Following PCR amplification, the PARP6^WT^ sequence corresponding to PARP6^ΔCRD^ was removed and replaced with the synthesized PARP6^ΔCRD^ gene fragment. All PARP6 expression constructs were verified for correct sequences by DNA sequence analysis.

### PARP6 immunoprecipitation auto-MARylation activity assay

PARP immunoprecipitation auto-MARylation activity assays were performed as previously described^8^, but with slight modifications. PARP6 ADP-ribosyltransferase activity was tested by transfecting HEK293T cells with PARP6^WT^, PARP6^Y487A^, and PARP6^ΔCRD^ expression plasmids containing an N-terminus GFP epitope. HEK293T cells plated on 6-well plates (35 mm well^−1^, Greiner Bio-One) were transfected with 3 μg plasmid per well using CalPhos^TM^ mammalian transfection kit. Cells were collected 24 h post-transfection and lysed in 250 μL well^−1^ cytosolic lysis buffer (CLB: 50 mM HEPES [pH 7.4], 150 mM NaCl, 1 mM MgCl_2_, 1 mM TCEP, 1% Triton X-100) with cOmplete^TM^ protease inhibitors. Lysates were centrifuged at 14,000× rpm for 10 min at 4 °C and supernatants were transferred to new sample tubes.

GFP-Trap®_MA magnetic agarose beads (Chromotek; 50 μL suspended bead slurry) were added to lysates for 1 h at 4 °C with constant rotation to immunoprecipitate GFP-PARP6. Following removal of supernatant, beads were washed twice with CLB, three times with CLB + 500 mM NaCl, and once with PARP reaction buffer (PRB: 50 mM Tris-HCl [pH 7.5], 50 mM NaCl, 0.5 mM T CEP, 0.1% Triton X-100) with cOmplete^TM^ protease inhibitors for 5 min per wash. Prior to ADPr labeling, beads were treated with 3 μM velaparib and 100 μM ATP for 30 min at 25 °C/650 rpm. 200 μM 6-alkyne-nicotinamide adenine dinucleotide (6-a-NAD^+^; 50 μL volume) in PRB was added to beads and incubated for 1 h at 25 °C/650 rpm. Following removal of 6-a-NAD^+^, beads were washed twice with PRB for 5 min per wash. Click reaction mixture (1 mM CuSO_4_, 1 mM TCEP, 100 μM TBTA, 100 μM biotin-azide; 25 μL volume) was added to beads and incubated for 1 h at 25 °C/650 rpm. Following removal of click reaction mixture, Laemmli sample buffer with 5% β-mercaptoethanol (30 μL volume) was added to beads. Samples were heated at 95 °C for 5 min and supernatants were transferred to new sample tubes. Samples were resolved by SDS-PAGE and transferred onto 0.45 μm PVDF membranes (BioTrace™, Pall Corp). Membrane blots were blocked with 5% milk-TBST for 1 h at room temperature. Blots were probed for PARP6 expression with anti-GFP primary antibody (chicken polyclonal, Abcam clone ab13970, 1:5000) for 2 h at room temperature and HRP-conjugated goat anti-chicken IgG secondary antibody for 1 h at room temperature. To detect PARP6 auto-MARylation activity, blots were probed for biotinylated proteins with HRP-conjugated streptavidin antibody (Jackson ImmunoResearch, 0.4 μg mL^−1^) for 1 h at room temperature. ECL HRP substrate (SuperSignal^TM^ West Pico, ThermoFisher) was added to detect protein targets by chemiluminescence. Blots were imaged for chemiluminescent signal on a ChemiDoc MP system (Bio-Rad).

### *In Utero* Electroporation

Surgical procedures for *in utero* electroporation were performed as previously described^19^. Time-pregnant rats were anesthetized with isofluorane. The abdomen was cleaned with 70% ethanol and a midline incision was made to expose the uterine horns/sacs. 2 μl of plasmid DNA (2 mg ml^−1^) prepared with FastGreen dye (Sigma) was injected into the right lateral ventricle of E19 embryos using a glass pipette pulled from thin-walled capillary glass (TW150F-4; World Precision Instruments) and a Picospritzer III microinjection system (Parker Hannifin). Following injection, each embryo within its uterine sac was positioned between tweezer-type electrodes (CUY650P10; Sonidel) and five square electric pulses (50 V; 100 ms; 1 s intervals) were passed using an electroporator (CUY21; Sonidel). After electroporation, the uterus was placed back into the abdominal cavity of the pregnant rat. The abdominal wall and skin were sutured closed to allow the embryos to develop to full term.

### *In Vivo* Tissue Processing

At P14, pups were anesthetized with 250 mg kg^−1^ tribromoethanol (i.p. injection) and transcardially perfused with 0.9% saline followed by 4% paraformaldehyde (PFA) (Electron Microscopy Sciences). Brains were post-fixed in 4% PFA for 24 h at 4 °C. Fixed brains were cut into 100 μm-thick coronal slices by vibratome sectioning (Leica). Sections were incubated with 2 μg mL^−1^ Hoechst-33258 for 15  min at room temperature. Following three 100 mM tris-saline washes, sections were mounted onto glass slides, allowed to dry, and coverslipped using ProLong Gold® antifade mountant (Life Technologies).

### Immunofluorescence and Immunostaining

DIV7 or DIV12 primary hippocampal neurons were fixed in 4% PFA and 4% sucrose in PBS (pH 7.4) for 20 min at room temperature. Fixed neurons were washed three times with PBS, washed once with distilled water, and mounted on a glass microscope slide (Fisher Scientific) with ProLong Gold® with DAPI antifade mountant.

For immunostaining, fixed neurons were permeabilized with 0.2% Triton X-100 in PBS for 5 min and washed three times with PBS. Neurons were then blocked with 10% HS/2% BSA blocking solution for 1 h at room temperature. Samples were incubated with primary antibodies in blocking solution for 24 h at 4 °C, washed three times with PBS, and incubated with Alexa Fluor-conjugated secondary antibodies in blocking solution (Molecular Probes, Jackson ImmunoResearch) for 1 h at room temperature. Coverslips were washed three times with PBS, once with distilled water, and mounted on a glass microscope slide with ProLong Gold with DAPI.

### Image Acquisition and Data Analysis

Fluorescent images of primary neurons were acquired on a Zeiss Apotome widefield microscope (Carl Zeiss, Inc.). For Sholl analysis, neurons were imaged with a 20x objective lens at a single z-section to fully capture the dendritic tree. Dendritic complexity was analyzed at 25 μm increments from the cell soma using Fiji software with Concentric Circles plugin (ImageJ). For higher-magnification visualization of dendritic regions, neurons were imaged with a 40× (whole-cell image) or 63× (dendritic image) oil-immersion objective lens through multiple z-sections (1.5 μm z-stack). Z-stack images were represented as 2-dimensional orthogonal projections using ZEN imaging software (Carl Zeiss, Inc.).

Fluorescent images of CA1 neurons from *in utero* electroporated coronal brain slices were acquired on a Yokogawa CSU-10 spinning disk confocal head mounted on a Nikon TE2000 inverted microscope. The CA1 region of the hippocampus was located using Hoechst counterstain. CA1 neurons were imaged with a 60× oil-immersion objective with a z-stack at 0.5 μm intervals to obtain the entire thickness of the dendrite in the 100 μm section. Dendritic complexity in CA1 neurons was analyzed by Sholl analysis at 25 μm increments from the cell soma using Fiji software with Concentric Circles plugin.

All graphs and statistical comparisons were generated using GraphPad Prism. Statistical analyses were performed using the two-tailed paired t-test or one-way ANOVA followed by Tukey’s HSD test. All data are presented as mean ± SEM.

## Additional Information

**How to cite this article**: Huang, J. Y. *et al.* PARP6 is a Regulator of Hippocampal Dendrite Morphogenesis. *Sci. Rep.* **6**, 18512; doi: 10.1038/srep18512 (2016).

## Supplementary Material

Supplementary Information
