# Supplementary material for: PARP6 is a Regulator of Hippocampal Dendritic Morphogenesis
Source: Sci Rep. 2016 Jan 4;6:18512. doi: 10.1038/srep18512 (PMC4698753; doi:10.1038/srep18512)
Supplement: Supplementary Information [file srep18512-s1.pdf]

**Supplementary Figures and Tables for**:

## PARP6 is a Regulator of Hippocampal Dendritic Morphogenesis

Jeffrey Y. Huang[1], Kang Wang[2], Anke Vermehren-Schmaedick[1], John P. Adelman[2], and Michael S. Cohen[1*]

[1]Department of Physiology and Pharmacology and [2]Vollum Institute, Oregon Health & Science University, Portland, Oregon 97210, United States

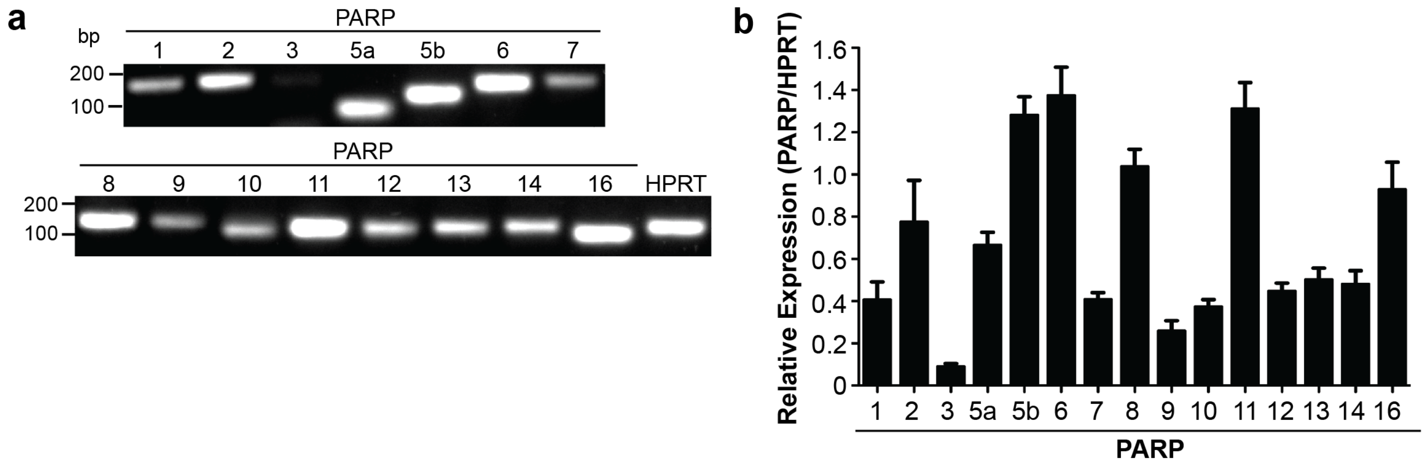


**Supplementary Figure 1. PARP6 is the most abundant PARP in E18 rat hippocampal neurons.**

(**a**) PARP family-wide mRNA expression profiling. RT-PCR was performed on cDNA from E18 rat hippocampal neurons. PARP-specific primer sequences used for RT-PCR are shown in **Supplementary table 1**. Hypoxanthine phosophoribosyltransferase (HPRT) served as a control.

(**b**) Quantification of results shown in **a**. Data were obtained from three independent experiments. Error bars represent SEM.

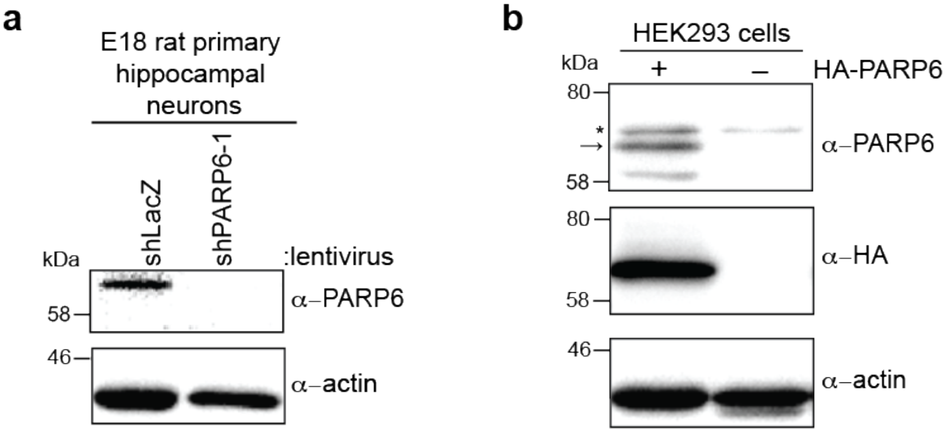


**Supplementary Figure 2. Specific detection of PARP6 using an anti-PARP6 antibody.**

(**a**) Validation of a commercially available PARP6 antibody using lentiviral mediated shRNA-based knockdown. E18 rat hippocampal neurons were transduced on day *in vitro* 0 (DIV0) with lentiviruses expressing either an shRNA targeting PARP6 (shPARP6-1) or LacZ (shLacZ). Neurons were harvested on DIV7 and proteins were resolved by SDS/PAGE and detected by Western blot with anti-PARP6 (Sigma; HPA026991) and anti-actin antibodies.

(**b**) Sigma anti-PARP6 detects HA-PARP6 overexpressed in HEK 293T cells. HEK 293T cells were transfected with HA-PARP6. Proteins were resolved by SDS/PAGE and detected by Western blot with anti-PARP6 (Sigma; HPA026991), anti-HA, and anti-actin antibodies. →denotes expected size of HA-PARP6 protein (~69 kDa), *denotes putative higher-molecular weight full-length splice variant of PARP6 protein (~71 kDa).

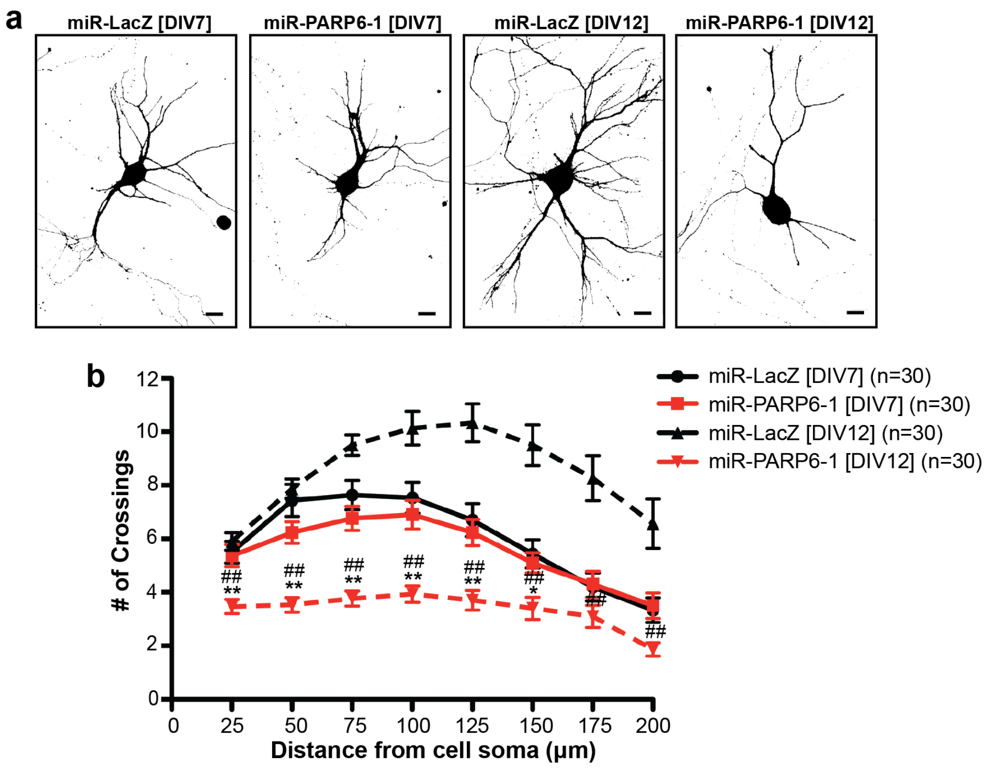


**Supplementary Figure 3. PARP6 knockdown reduces dendritic growth and maintenance.**

(**a**) E18 primary rat hippocampal neurons were transfected with miRNA-based knockdown plasmids on DIV6 and fixed on either DIV7 or DIV12. Shown are representative binary images generated from fluorescent images. Scale bar, 20 μm.

(**b**) Quantification of results in **a** using Sholl analysis. Error bars represent SEM. *p<0.05, **p<0.001 (one-way ANOVA followed by Tukey's HSD test) compared to miR-LacZ [DIV7]; ##p<0.001 (one-way ANOVA followed by Tukey's HSD test) compared to miR-LacZ [DIV12].

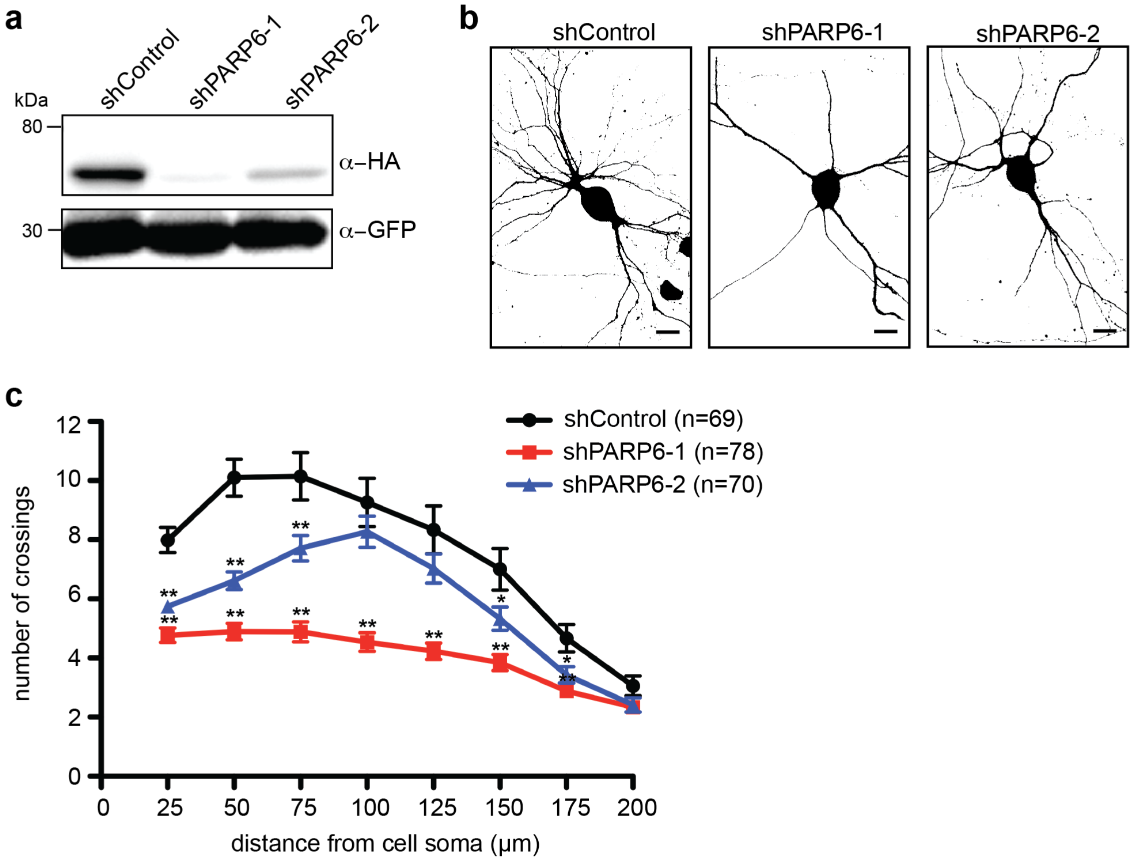


**Supplementary Figure 4. PARP6 regulates dendritic complexity in primary hippocampal neurons.**

(**a**) shRNA-based plasmids effectively knockdown PARP6 protein levels. HEK 293T cells were co-transfected with HA-PARP6 and GFP-expressing shRNA-based knockdown plasmids targeting rat PARP6 (shPARP6-1 or 2) or a non-targeting control (shControl). Proteins were resolved by SDS/PAGE and detected by Western blot with anti-HA and anti-GFP antibodies.

(**b**) Knockdown of PARP6 using shRNA-based plasmids decreased dendritic complexity. E18 primary rat hippocampal neurons were transfected with shRNA-based knockdown plasmids on DIV7 and fixed on DIV12. Shown are representative binary images generated from fluorescent images. Scale bar, 20 μm.

(**c**) Quantification of results in **b** using Sholl analysis. Error bars represent SEM. *p<0.05, **p<0.001 (one-way ANOVA followed by Tukey's HSD test) compared to shControl.

```
                   Cysteine-rich Domain (CRD)
mPARP6   EQRIPTLNEYCVVCDEQHVFQNGSMLKPAVCTRELCVFSFYTLGVMSGAAEEVATGAEVV
hPARP6   EQRIPTLNEYCVVCDEQHVFQNGSMLKPAVCTRELCVFSFYTLGVMSGAAEEVATGAEVV
rPARP6   EQRVPTLNEYCVVCDEQHVFQNGSMLKPAVCTRELCVFSFYTLGVMSGAAEEVATGAEVV
xPARP6   EQRLPTLNEYCVVCDEQHVFQNASMLKPAVCTRELCVFSFYTLGVMSGAAEEVATGAEVV
dPARP6   EQRIPTLNDYCVVCDEQHVFQNGSMLKPAVCTRELCVFSFNTLGVMSGAAEDVATGAEVV
                  266                      291
```

**Supplementary Figure 5. Alignment of PARP6 orthologues demonstrates conservation of the cysteine-rich domain (CRD).** mPARP6 = Mus musculus PARP6 (NM_001205239); hPARP6 = Homo sapiens PARP6 (NM_020214); rPARP6 = Rattus norvegicus PARP6 (NM_001106828); xPARP6 = Xenopus tropicalis PARP6 (NP_001090857); dPARP6 = Danio rerio PARP6 (XP_009296376).

**Supplementary Table 1: PARP-specific primer sequences for endpoint-PCR expression profiling**

| PARP | Forward Primer Sequence | Reverse Primer Sequence | Amplicon Length |
|---|---|---|---|
| 1 | TCTACTTTGCTGATATGGTGTCC (bp 2746-2768) | TGGGTAACTTGCTGATGTGAG (bp 2889-2869) | 144 |
| 2 | GAAATTGCCCTTAAGCTGGTG (bp 980-1000) | TGGGTAGACTGTAAGTACTGGG (bp 1128-1107) | 149 |
| 3 | CTCGGAGAACAGCAAGTCAG (bp 1349-1368) | GGGTGGACTCTTCAAGCTG (bp 1490-1472) | 142 |
| 5a | CACCGCCACAAGTTAATCAAG (bp 3218-3238) | AAGGTCAGATACGGATTGGTTC (bp 3291-3270) | 74 |
| 5b | AGCGAGAACAGATCACCTTG (bp 2684-2703) | ACCCCTTTAATGAGCCTGTG (bp 2789-2770) | 106 |
| 6 | CTCCGTGTCCATCAGAGAATATG (bp 645-667) | ACCGTAGCCTCAACACAATAG (bp 781-761) | 137 |
| 7 | GTATGCCTGTCCTGATTCCTG (bp 586-606) | TCTGCTACTGTGCCATTGATG (bp 727-707) | 142 |
| 8 | ATGTTAGCCTTCAACCCCAG (bp 2158-2177) | CAGAAGTGGATGAGCAAGGG (bp 2307-2288) | 147 |
| 9 | TGGGTGTAGAAGTGGGAGAG (bp 1761-1780) | TTGAACTTGTCTGGAGCTGAG (bp 1910-1890) | 150 |

| | | | |
|---|---|---|---|
| 10 | GAAATGGTGCTGTCAATGGAG (bp 1210-1230) | CCGAAAGCCAGTCACATCTAC (bp 1335-1315) | 126 |
| 11 | TGACTGGAGAATCAATGGTGTG (bp 956-977) | AAGCTGACCCCATGAATCTG (bp 1093-1074) | 138 |
| 12 | TGTTTGTTAAGACCAAGCACAGA (bp 3011-3033) | AGGCCACTATGCTCCTCTGA (bp 3152-3133) | 142 |
| 13 | CGGTGGCAGATGTTCGTATAT (bp 1840-1860) | GGTAGGAGTCACAGGTCATTTTC (bp 1984-1962) | 145 |
| 14 | GACATTGGTATCTGCCGTAGG (bp 5387-5407) | TCAAGTGGTTGGCTCTCAAG (bp 5535-5516) | 149 |
| 16 | CCGCCTAGAGAACTTCCATTC (bp 582-602) | CCGTGAGGACTATAAATGAGGG (bp 710-689) | 129 |

**Supplementary Table 2: PARP6 RNAi sequences**

| **RNAi constructs** | **Target Sequence** | **Species Specificity** |
|---|---|---|
| miR-LacZ | GACTACACAAATCAGCGATTC | N/A |
| miR-PARP6-1 | TTGCACCATGAAGAACCCAAA (bp 1125-1145) | Mouse, Rat |
| miR-PARP6-2 | GGGCTCATATTTGGAAATCAA (bp 1674-1694) | Mouse, Rat |

| | | |
|---|---|---|
| miR-PARP6-3 | CTACACTAAACTGCAGCTGCA (bp 1959-1979) | Rat |
| shControl | ACTACCGTTGTTATAGGTGTC | N/A |
| shPARP6-1 | TTGCACCATGAAGAACCCAAA (bp 1125-1145) | Mouse, Rat |
| shPARP6-2 | GATGACCCAGGGCTCATATTT (bp 1665-1685) | Mouse, Rat |
